# Chronic Pravastatin but Not Atorvastatin Treatment Impairs Cognitive Function in Two Rodent Models of Learning and Memory

**DOI:** 10.1371/journal.pone.0075467

**Published:** 2013-09-10

**Authors:** Sarah A. Stuart, James D. Robertson, Neil V. Marrion, Emma S. J. Robinson

**Affiliations:** School of Physiology and Pharmacology, University of Bristol, Bristol, United Kingdom; Alexander Flemming Biomedical Sciences Research Center, Greece

## Abstract

Statins are some of the most commonly prescribed drugs and are used to reduce blood cholesterol. Recent evidence suggests that, in some patients, they may adversely influence cognitive function including causing memory impairments. These clinical observations have led to statin prescriptions now including a warning about possible cognitive impairments. In order to better understand the relationship between statin treatment and cognitive function, studies in animals are needed. The present study investigated the effects of chronic treatment with two statins, pravastatin and atorvastatin, in two rodent models of learning and memory. Adult rats were treated once daily with pravastatin (10mg/kg, orally) or atorvostatin (10mg/kg, orally) for 18 days. Before, during and after treatment, animals were tested in a simple discrimination and reversal learning task. On the last day of treatment and following one week withdrawal, animals were also tested in a task of novel object discrimination. Pravastatin tended to impair learning over the last few days of treatment and this effect was fully reversed once treatment ceased. In the novel object discrimination task, pravastatin significantly impaired object recognition memory. No effects were observed for atorvostatin in either task. These data suggest that chronic treatment with pravastatin impairs working and recognition memory in rodents. The reversibility of the effects on cessation of treatment is similar to what has been observed in patients, but the lack of effect of atorvostatin suggests that lipophilicity may not be a major factor influencing statin-induced cognitive impairments.

## Introduction

The class of drugs known as statins (3-hydroxy-3-methylglutaryl-CoA [HMG-CoA] reductase inhibitors) are commonly prescribed to lower cholesterol in patients suffering from, or at risk of, cardiovascular complications [1]. They are generally well tolerated in patients [2] and as such there is some support for the extension of statin treatment as a preventative therapy in people with healthy levels of cholesterol [3]. Almost a quarter of the unesterified cholesterol present in the body is located within the central nervous system, and it is integral to many CNS functions including within the myelin sheath and synapse formation [4–6]. It is therefore possible that disrupting the balance of cerebral cholesterol metabolism could adversely affect brain function. There is also emerging evidence that statins can cause cognitive impairments in some patients and in 2012, the information associated with stain prescriptions was updated to include possible adverse cognitive effects, including memory problems and confusion [7].

Observational studies have indicated that chronic statin therapy may reduce the risk of developing dementia and Alzheimer’s disease (AD), as well as potentially reducing the rate of cognitive decline associated with the conditions [8–10]. Nevertheless, the more recent LEADe study (Lipitor’s Effect in Alzheimer’s Dementia) reported no clinical benefit of atorvastatin as a treatment for mild or moderate AD [11]. Despite the evidence for positive effects of statin treatment on cognitive function in AD, published case reports describe that some non-AD patients experience impairments of short term memory and amnesia during statin therapy that resolve with cessation of treatment [12,13]. Muldoon et al., (2004) [14] in a randomised trial with healthy adults, found that treatment with simvastatin was associated with decreased performance on some neuropsychological tests compared with the placebo group. Furthermore, a review of patients who self-reported memory or other cognitive problems associated with statin therapy, found evidence supporting a relationship between statin potency and significant negative impact on quality-of-life [15].

The available animal studies regarding statin effects on cognition have mostly been carried out in animal models of AD or traumatic brain injury. Some of these studies have shown statins to improve performance in learning and memory paradigms such as the Morris water maze [16,17]. In addition, simvastatin enhances long-term potentiation (LTP) in hippocampal slices *in vitro* and stimulates hippocampal neurogenesis and expression levels of neurotrophic factors such as brain derived neurotrophic factor (BDNF) and vascular endothelial growth factor (VEGF) [18–20]. With the exception of a recent study by Baytan et al. (2008) [21], demonstrating a statin-induced impairment of spatial memory in naïve rats, data regarding the effects of statin treatment *in vivo* without prior manipulation of cognitive function is lacking. Since cholesterol is essential in the myelination of neurons and other neuronal functions, it has been proposed that excessive inhibition of cholesterol synthesis could lead to adverse cognitive effects [22]. Recent research has also shown that disruption of the cholesterol-rich membrane microdomains known as ‘lipid rafts’ alters cell signalling pathways that might predict cognitive impairment [23]. A limited number of studies have investigated brain lipid composition in animals following statin treatments. In a study by Vecka et al. (2004) [24], four different statins, including pravastatin were found to alter brain lipid composition following 6 weeks of chronic dosing. Other studies have shown that simvastatin reduces brain cholesterol in guinea pigs [25] whilst simvastatin but not pravastatin was found to affect brain cholesterol synthesis in mice [26].

In the present study, we investigated the effects of two statins, the hydrophilic compound pravastatin and the lipophilic compound, atorvastatin on learning and memory in normal adult rats. The drugs were administered chronically for 18 days during which animals were tested in two models of learning and memory: a simple discrimination and reversal learning (SDRL) paradigm, and a novel object discrimination (NOD) task. In clinical populations, any cognitive impairment associated with statin treatment has been reported to reverse upon cessation of treatment. Whilst previous animal studies have investigated the effects of chronic statin treatment on lipid levels in rodents [24–26], studies into their cognitive effects and reversibility are limited. Therefore, we decided to focus this study on assessing the reversibility of any deficit as opposed to euthanizing the animals at the end of treatment to assess circulating cholesterol levels.

## Methods

### Ethics statement

All procedures were conducted in accordance with the requirements of the UK Animals (Scientific Procedures) Act 1986 and in accordance with local institutional guidelines and under a Home Office project license (PPL 30/2443). Experiments were conducted and are reported in line with the ARRIVE guidelines [27].

### Subjects

**Figure 1:**
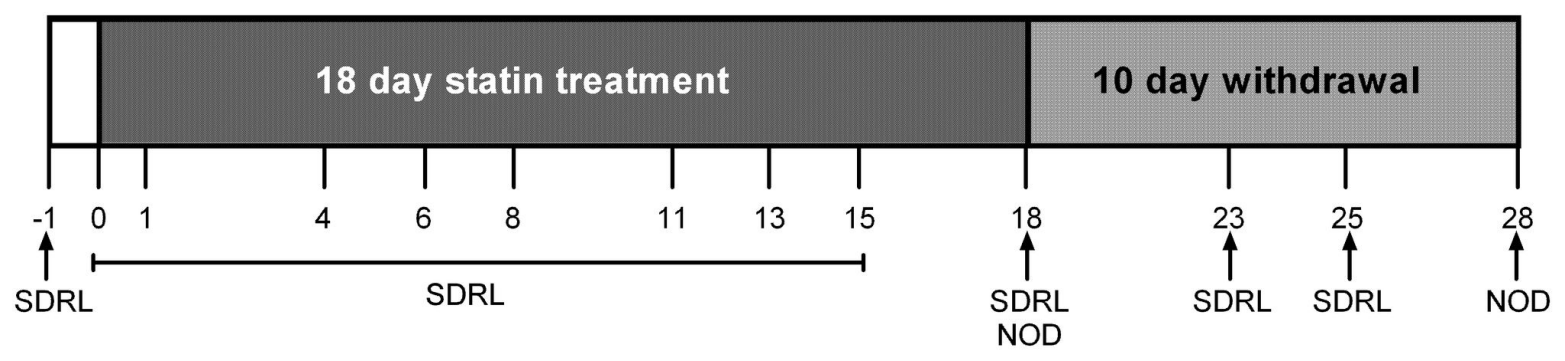
Timeline illustrating the study design. Animals received once daily treatment with statin or vehicle orally between 4 and 5 pm for a total of 18 days. Simple discrimination and reversal learning was tested the day before drug treatment commenced (-1), during treatment on days 0, 1, 4, 6, 8, 11, 13, 15, and 18, and post-treatment on days 23 and 25. Novel object discrimination tests were carried out during treatment on day 18 and post-treatment on day 28.

The animals used were 24 male Lister-hooded rats weighing approximately 400-500g (approximately 7 months old) at the start of training (Harlan, UK), housed in groups of four under specific pathogen free temperature-controlled conditions and a 12:12h reverse light-dark cycle (lights off at 0700h). Animals were housed in standard laboratory cages with sawdust, paper bedding and cardboard tubes. They were cleaned out once per week, with bedding and tubes replaced as necessary. They were maintained at approximately 85% of their free-feeding weight by restricting access to laboratory chow (Purina, UK) to approximately 18g per rat per day with the animals individual weights recorded at least once a week. Water was provided *ad libitum*. All dosing was carried out in the animal’s home cage and all behavioural testing was carried out between 0900 h and 1700 h during the animals’ active phase. Animals were randomly assigned to one of three treatment groups (n=8 animals/grp) and all experiments were carried out with the experimenter blind to treatment. An overview of the experiment is given in Figure 1.

### Apparatus

The animals were tested in a Perspex arena (40 cm^2^) for both behavioural tasks in a separate room from the holding room. In the simple discrimination and reversal learning (SDRL) task the substrates e.g. bedding, sawdust, sand, cloth, perlite etc, are placed in glazed pottery bowls (10 cm diameter) and presented in a pseudo-random order in the left or right position. This prevented the rats using spatial cues to select the correct substrate and food reward. A 10ml glass cylinder, 100ml glass conical flask and glass 100ml beaker were used as objects in the novel object discrimination (NOD) task. All objects were cleaned with 70% ethanol between trials.

### Experiment 1: Simple discrimination and reversal learning

The rats were first habituated to the test arena and trained to dig in two bowls filled with sawdust to obtain a quantity of reward pellets (45mg precision pellets, Sandown Scientific UK). On the first day of training, one pellet was buried within the sawdust, and another three placed on top to encourage digging. Each rat was individually placed in the test arena facing the two bowls and given 10mins to explore, with the trial being terminated once all pellets had been consumed and the rat had left the second bowl. If all the pellets were not found within the 10 minute period, the rat was removed and the bowls re-baited. On the consecutive training day, a single reward pellet was buried in each bowl and the rats were allowed 5mins to explore both bowls. Training was complete once each rat was able to find the pellet in each bowl on 12 consecutive trials.

Each discrimination session consisted of individual trials in which the rat was required to choose between one of the two bowls, containing different digging mediums, to locate a reward pellet. In each of these trials, one of the bowls contained a ‘reward-paired’ substrate and the other contained a different, ‘unrewarded’ substrate. The pairs of substrates used were changed for each day of testing. In the blank substrate, the equivalent number of sugar pellets was crushed into the bowl to avoid discrimination based on odour. The bowls were positioned (left or right) using a pseudo-random order to avoid spatial learning. The rat was placed in front of the two bowls and allowed to dig in one of the two bowls. Once the animal began to dig, the other bowl was removed from the test arena. Digging in the reward-paired substrate was recorded as a correct trial, and digging in the blank substrate was recorded as an incorrect trial. The latency to dig was also recorded for each trial and the session was completed once the rat reached a criterion of 6 consecutive correct trials (the probability of making a 6 consecutive correct choices by chance being 0.015). There was no maximum for the number of trials and animals were tested until they reached criterion. The reversal phase followed the same protocol, but the location of reward was switched such that the previously blank substrate became the reward-paired substrate. The reversal phase was completed once the rat reached a criterion of 6 consecutive correct trials. Animals were tested on the day before treatment started, during treatment on days 0, 1, 4, 6, 8, 11, 13, 15 and 18, and after treatment on days 23 and 25 (Figure 1).

### Experiment 2: Novel Object Discrimination Task

Each rat sequentially received two consecutive 5 min object exploration trials separated by a 4 h inter-trial interval (ITI) in the home cage. Rats were exposed to two objects during the first (familiarisation) trial, and one of the objects was selected at random and replaced with a third, novel object in the second (choice) trial. During the two trials exploration of each object, defined as sniffing, licking, chewing, or having moving vibrissae while directing the nose toward and ≤1 cm from the object, was recorded separately using stopwatches. Sitting on an object in the absence of any directed interest was not regarded as exploratory activity, but rarely occurred. The objects and test arena were wiped with 70% (v/v) ethanol between trials to reduce olfactory cues. The discrimination (D) ratio was calculated as time spent exploring the novel object compared with the familiar object relative to the total time spent exploring all objects, according to the formula: (t [novel]-t [familiar]) / (t [novel] +t [familiar]) *100. Novel object discrimination testing was carried out once on day 18 of treatment, at least 2 hrs after the SDRL test had been competed (Figure 1).

### Post drug treatment

The rats were withdrawn from drug treatment after task completion on day 18. After 5 and 7 days of withdrawal (study days 23 and 25), the animals were re-tested in the SDRL tasks, and re-tested in the NOD task after 10 days of withdrawal (study day 28).

### Drugs

Pravastatin (pravastatin sodium tablet, Teva UK) and atorvastatin (atorvastatin calcium tablet, Pfizer, UK) were obtained from a local pharmacy and crushed using a pestle and mortar and suspended in a vehicle of strawberry milk shake (Yazoo™). During pre-treatment training, rats were trained to take milk shake (0.5ml) from a 1ml syringe. On each day of treatment, drugs were freshly prepared and administered orally at a dose volume of 1ml/kg. Drugs were administered between 4 and 5pm daily, at least 2 hrs after testing and 16hrs before the next behavioural experiment. The choice of dose was based on previous behavioural studies using statin treatments ( [28] 1, 5mg/kg [21]; 10 and 30 mg/kg [29]; 10mg/kg). Clinical trial doses of pravastatin and atorvostatin are in the region of 40mg and 10mg respectively, in line with the higher relative potency of atorvastatin [30]. These clinical doses have been reported to have similar efficacy in terms of reductions in plasma cholesterol [31].

### Statistical Analysis

Graphs were constructed using Graphad Prism 5.0 (Graphpad Software, USA). Choice latency and trials to criterion data in the SDRL task were analysed using a repeated measures ANOVA with DAY as the within-subject factor and TREATMENT as the between-subjects factor. Based on the case studies in patients and *in vitro* experiments, our *a priori* hypothesis predicted that the effects of the statin treatment would increase over the course of the chronic treatment. Because the number of data points recorded over the whole period of the study had the potential to limit detection of any changes developing during the latter part of the study, we also analysed the results from the last test day using a one-way ANOVA with Dunnet’s test *post-hoc*. D ratio data and total exploration time were analysed using a one-way ANOVA with TREATMENT as the between-subjects factor, followed by *post hoc* Dunnet’s. Where a data point appeared to lie outside the normal range, Dixon’s Q test was used to determine whether this was an outlier.

## Results

### Body weight

**Figure 2:**
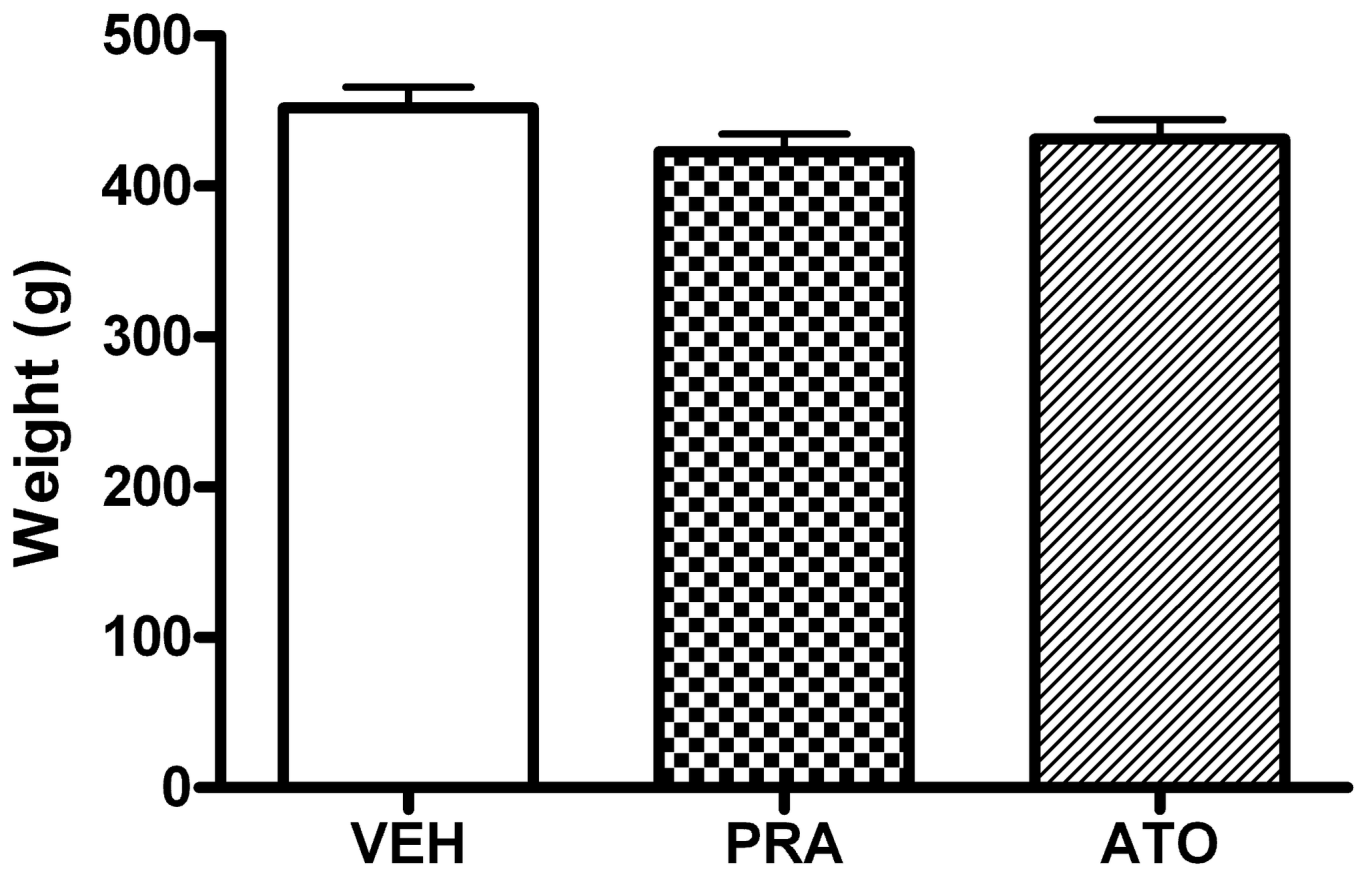
Effects of chronic treatment with statins on body weight in normal rats. Body weight was recorded at the end of the study. There was no significant difference in group body weight suggesting treatments did not adversely affect the animals’ overall health. Data shown as mean body weight ± s.e.m.

There was no significant effect of treatment on group body weight recorded at the end dosing, day 18 (Figure 2).

### Simple discrimination (SD) and reversal learning (RL) tasks

**Figure 3:**
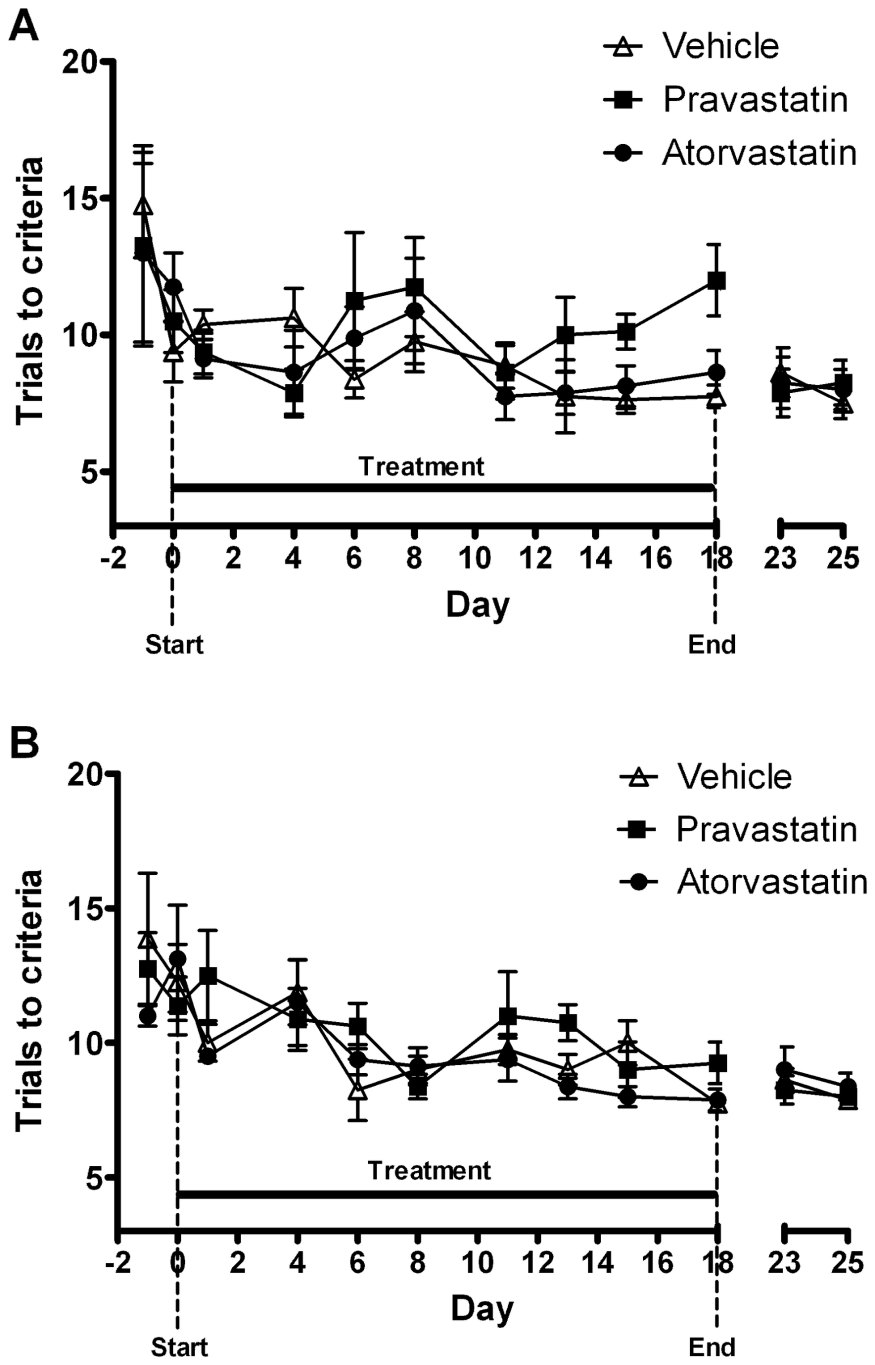
Effect of chronic statin treatment (10mg/kg p.o.) on learning in simple discrimination (A) and reversal learning tasks (B) in normal rats. All treatment groups demonstrated a significant learning effect in both tasks, showing a significant decrease in trials to criteria across the study period. There was no significant main effect of statin treatment across the study period compared to vehicle-treated animals. Data shown as mean trials to criteria ± s.e.m., n = 8 animals per group. Period of drug treatment is denoted by horizontal line.

**Figure 4:**
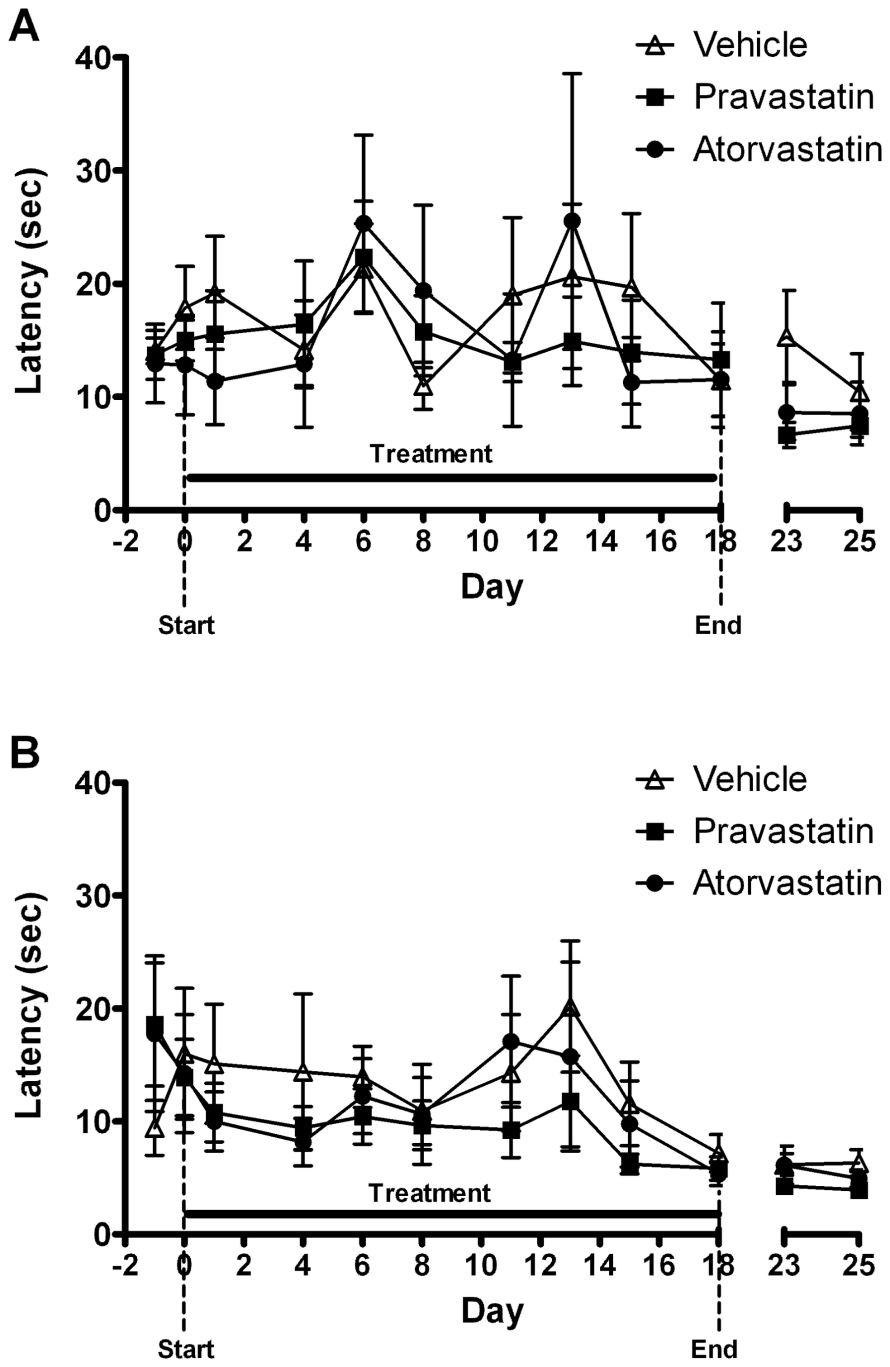
Effect of chronic statin treatment (10mg/kg p.o.) on trial latency in simple discrimination (A) and reversal tasks (B) in normal rats. All treatment groups demonstrated a significant decrease in response latency as the study progressed but there was no significant effect of statin treatment on response latency before, during or after treatment compared to vehicle-treated animals. Data shown as mean latency ± s.e.m. Period of drug treatment is denoted by horizontal line.

**Figure 5:**
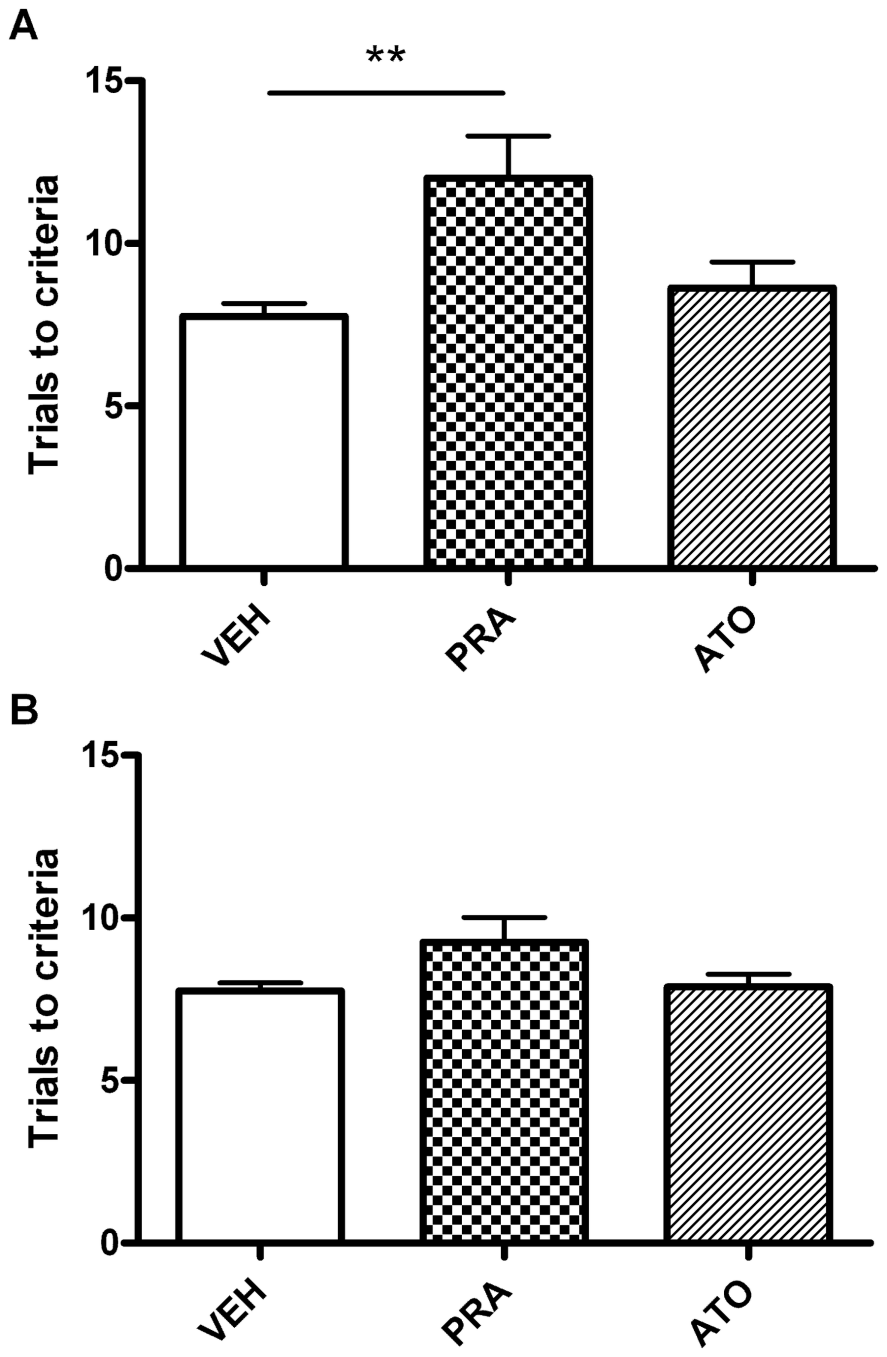
Effect of chronic statin treatment (10mg/kg p.o.) on learning in a simple discrimination (A) and reversal task (B) on the final day of treatment. On the final day of treatment, pravastatin significantly impaired learning ability in the simple discrimination task as reflected by an increase in trials to criteria compared to controls (**p<0.01; n=8/group). There was a trend towards an impairment in reversal learning following pravastatin treatment (p=0.09; n=8/group). Atorvastatin had no effect on learning in either task. Data shown as mean trials to criteria ± s.e.m.

There was no significant effect of *treatment* on the number of trials to criteria across the full study period (Figure 3, Table 1), however there was a significant effect of *day* in both the simple discrimination (ANOVA: F_11,231_ = 3.91, P<0.0001) and reversal learning (ANOVA: F_11,231_ = 6.92, P<0.0001) tasks. All treatment groups showed a significant decrease in the number of trials to criteria across the study, demonstrating an improvement in task performance with repeated exposure. There was a significant effect of day on the response latency in both tasks (SD:F_11, 231_ = 4.46, P<0.0001; RL: F_11,231_ = 4.57, P<0.0001, Figure 4), with all groups responding more quickly as the study progressed. In contrast, there was no significant effect of drug on response latency before, during or after treatment. Further analysis of the last day of drug treatment showed that animals treated with pravastatin required more trials to reach criteria during the simple discrimination task on the final day of drug treatment (day 18) compared with the vehicle-treated group (one way ANONA F_2,23_=5.99, p=0.009, p<0.05 pravastatin versus vehicle, Figure 5). There was no significant effect of pravastatin on reversal learning (one way ANONA F_2,23_=2.54, p=0.10). Latencies were not significantly different following 18 days of pravastatin treatment and atorvastatin did not significantly affect trials to criteria or latency in either task.

**Table 1:** Summary of the results from the simple discrimination and reversal learning experiment before, during and after chronic administration of control, pravastain or atorvostatin (one x daily for 18 days).

**Trials to criterion (6 consecutive correct responses)**								**Latency to dig**						
**Discrimination**	**Control**		**Pravastatin**		**Atorvastatin**			**Discrimination**	**Control**		**Pravastatin**		**Atorvastatin**	
**Day**	**Mean**	**sem**	**Mean**	**sem**	**Mean**	**sem**		**Day**	**Mean**	**sem**	**Mean**	**sem**	**Mean**	**sem**
***-1***	*14.75*	*1.93*	*13.25*	*3.66*	*13.00*	*3.26*		***-1***	*14.08*	*1.13*	*13.71*	*2.16*	*12.95*	*3.47*
**0**	9.38	1.10	10.50	1.15	11.75	1.25		**0**	17.86	3.67	15.00	1.79	12.84	4.40
**1**	10.38	0.53	9.38	0.80	9.13	0.69		**1**	19.19	5.00	15.55	3.82	11.40	3.83
**4**	10.63	1.07	7.88	0.88	8.63	1.53		**4**	14.14	3.13	16.41	5.61	12.93	5.59
**6**	8.38	0.68	11.25	2.49	9.88	1.16		**6**	21.34	3.95	22.35	4.94	25.33	7.82
**8**	9.75	1.10	11.75	1.81	10.88	1.93		**8**	10.99	2.08	15.77	3.20	19.39	7.53
**11**	8.88	0.83	8.63	0.98	7.75	0.86		**11**	18.98	6.87	13.08	1.73	13.26	5.85
**13**	7.75	1.33	10.00	1.38	7.88	0.79		**13**	20.62	6.42	14.92	3.92	25.56	13.03
**15**	7.63	0.50	10.13	0.64	8.13	0.74		**15**	19.70	6.50	13.97	4.60	11.30	3.94
**18**	7.75	0.41	12.00	1.31	8.63	0.80		**18**	11.48	3.21	13.29	5.02	11.54	4.21
***23***	*8.63*	*0.91*	*7.88*	*0.88*	*8.25*	*0.94*		***23***	*15.33*	*4.07*	*6.65*	*1.10*	*8.65*	*2.45*
***25***	*7.50*	*0.57*	*8.25*	*0.82*	*8.00*	*0.73*		***25***	*10.45*	*3.36*	*7.46*	*1.00*	*8.55*	*2.78*
**Reversal**	**Control**		**Pravastatin**		**Atorvastatin**			**Reversal**	**Control**		**Pravastatin**		**Atorvastatin**	
**Day**	**Mean**	**sem**	**Mean**	**sem**	**Mean**	**sem**		**Day**	**Mean**	**sem**	**Mean**	**sem**	**Mean**	**sem**
***-1***	*13.88*	*2.43*	*12.75*	*1.35*	*11.00*	*0.38*		***-1***	*9.43*	*2.43*	*18.60*	*5.44*	*17.79*	*6.89*
**0**	12.25	1.41	11.38	1.07	13.13	1.99		**0**	16.01	5.79	13.90	3.39	14.24	5.23
**1**	10.00	0.68	12.50	1.68	9.50	0.27		**1**	15.11	5.29	10.79	2.59	10.00	2.62
**4**	11.88	1.20	10.88	1.16	11.50	1.59		**4**	14.39	6.91	9.43	1.89	8.18	2.08
**6**	8.25	1.15	10.63	0.84	9.38	0.56		**6**	13.95	2.70	10.45	2.45	12.24	3.33
**8**	9.00	0.50	8.38	0.46	9.13	0.69		**8**	10.93	2.97	9.66	2.15	10.64	4.43
**11**	9.75	0.56	11.00	1.65	9.38	0.80		**11**	14.31	5.15	9.26	2.44	17.07	5.81
**13**	9.00	0.57	10.75	0.67	8.38	0.46		**13**	20.19	5.81	11.79	4.04	15.75	8.37
**15**	10.00	0.82	9.00	1.04	8.00	0.38		**15**	11.57	3.72	6.24	0.87	9.78	3.80
**18**	7.75	0.25	9.25	0.77	7.88	0.40		**18**	7.17	1.66	5.85	1.04	5.34	1.02
***23***	*8.63*	*0.42*	*8.25*	*0.53*	*9.00*	*0.85*		***23***	*6.19*	*0.95*	*4.29*	*0.50*	*6.14*	*1.69*
***25***	*7.88*	*0.23*	*8.00*	*0.27*	*8.38*	*0.50*		***25***	*6.33*	*1.17*	*3.95*	*0.28*	*4.95*	*0.79*

Italic results relate to test days pre- or post-treatment.

### Novel object discrimination task

**Figure 6:**
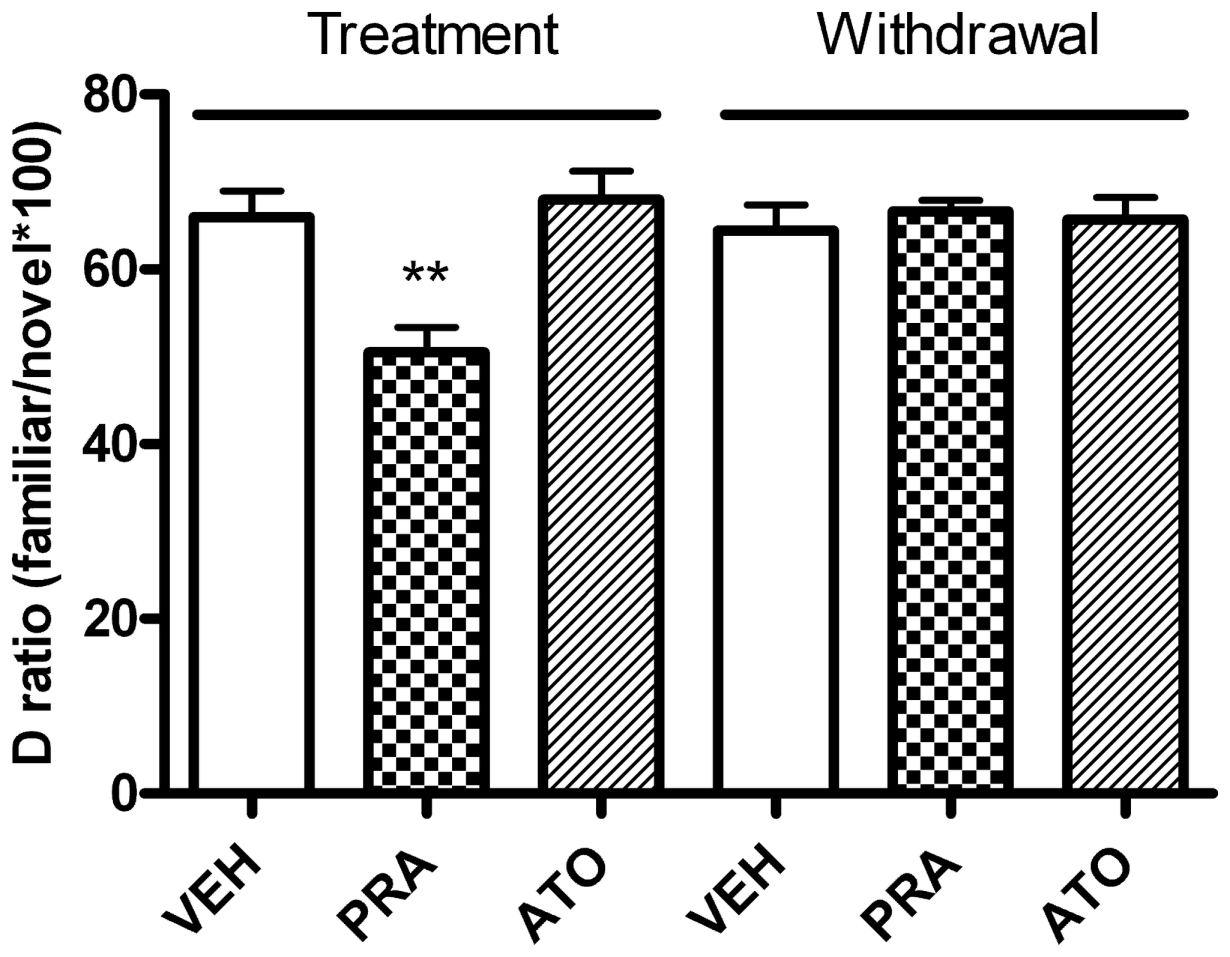
Effect of chronic statin treatment (10mg/kg p.o.) and withdrawal on exploration in a novel object discrimination (NOD) task in normal rats. Pravastatin, but not atorvastatin significantly impaired the animals’ ability to discriminate the novel from the familiar object after 18 days of treatment. Following 10 days withdrawal from treatment, there were no significant differences between the treatment groups. Data shown as mean D ratio ± sem, **p<0.01 vs. vehicle; n = 7-8/group (1 animal excluded from pravastatin group).

One rat in the pravastatin group was excluded from the analysis (Q_exp_ = 0.469 <Q_crit_=0.526 (CL 95%)). There was a significant effect of drug treatment on discrimination ratio in the novel object task (One way ANOVA, F_2,20_ = 5.17, p = 0.002). *Post hoc* testing revealed that animals treated with pravastatin showed an impaired ability to discriminate the novel from the familiar object (reflected as a decrease in D ratio) compared with the control group (Figure 6, Table 2). Atorvastatin treatment did not significantly affect performance in this task. Total exploration was not significantly affected by drug treatment suggesting this effect was specific to object discrimination rather than any non-specific effects on engagement in the task (One way ANOVA, F_2,20_ = 1.19, p = 0.33, Table 2). This effect was fully reversed following withdrawal from drug treatment with no significant differences observed between groups for discrimination (One way ANOVA, F_2,20_ = 0.54, p = 0.59) or total exploration time (One way ANOVA, F_2,20_ = 0.51, p = 0.60).

**Table 2:** Summary of the results from the novel object discrimination task at the end of treatment with control, pravastain or atorvostatin and following 10 days withdrawal from treatment.

**Novel Object Discrimination**								
**D ratio**	**Treatment day 18**			**One-way ANOVA**	**Post-drug day 10**			**One-way ANOVA**
**Mean**	64.7	50.5**	68.86	f[2,20]=8.96, p=0.002	63.98	67.49	64.66	f[2,20]=0.54, p=0.54
**sem**	3.11	2.89	3.61		3.31	1.09	2.67	
**Exploration time**								
**mean**	34.38	27.75	24.38	f[2,20]=1.26, p=0.31	28.94	26.95	24.34	f[2,20]=0.46, p=0.63
**sem**	5.919	4.956	1.511		2.539	3.38	3.658	
**n**	8	7	8		8	7	8	

** p<0.01 vs control, one-way ANOVA with Dunnett’s test *post hoc*

Data from one animal in the pravastatin group was excluded from the whole study as their D ratio data on treatment day 18 was an outlier

(Dixon’s Q-test, data point value 83.33)

## Discussion

In the current study we observed impaired performance in both tasks following treatment with pravastatin but not atorvastatin. Pravastatin significantly impaired simple discrimination learning on the last treatment day. A significant impairment in novel object discrimination was also observed following pravastatin but not atorvastatin treatment. The impairments developed after chronic treatment and, as observed in patients, were fully reversed when animals were re-tested following at least 5 days withdrawal from treatment. The adverse effects of pravastatin on memory is in keeping with Baytan et al. (2008) [21] who also found cognitive deficits arising from simvastatin treatment in normal rats in a test of spatial working memory. The results also support clinical observations that statin treatment can impair memory function in humans [12–14], and suggest that further investigations into the mechanisms underlying the effects observed are warranted.

The simple discrimination and reversal learning task uses a similar procedure to the attentional set shifting paradigm [32], where animals are trained to use specific cues to identify the bowl containing the food reward. In this study, different digging mediums were used as the cue and animals were tested repeatedly using the same procedure. On each test session, the animals were required to learn which digging medium was associated with the reward. Once the animal reached criteria for discrimination learning, the reward contingency was reversed and the number of trials taken to learn this new rule was recorded. Over the course of the study, all animals showed an improvement in task performance suggesting they were able to learn the underlying rule of the task over each successive test day. This effect was primarily observed over the first few sessions, but did continue through the initial phase of drug treatment. Initially, treatment with the statins did not induce any performance deficits. All groups exhibited a similar level of discrimination learning on each day with no effects on the number of trials to criteria for either SD or RL. The latency to make a choice was also not affected by treatment, suggesting that the drugs did not have any acute effects on motivation to perform the task or non-specific motor effects. There was also no effect of treatment on body weight between the treatment groups.

Although there was no overall effect of treatment on performance in the SD and RL task, the number of data points included in the ANOVA analysis makes it unlikely that changes induced towards the end of treatment would be detected. Based on our hypothesis that chronic treatment would result in the gradual development of a cognitive impairment, we analysed the results as a whole and on the last day of treatment separately. Although this analysis has its limitations, our findings suggest that pravastatin but not atorvastatin impaired SD and RL with a deficit in performance observed following 18 days of treatment. As a test of working memory, the results indicate that rats treated with pravastatin take longer to learn the correct digging medium. Previous studies using this type of test have shown that drugs that impair both attention and working memory can induce impairments in task performance [33,34]. The reversal phase requires cognitive flexibility where animals are required to disengage from the previously rewarded substrate and select the previously unrewarded substrate [35]. At this stage, the specific cognitive domain associated with the impairment we observed has not been elucidated. Clearly, additional studies using other models such as the 5-choice serial reaction time task [36], serial reversal learning task [37] and delayed matching/non-matching to sample task [38] will be required.

Novel object discrimination provides a test of recognition memory in rodents. Rodents are predisposed to investigate novelty and will bias their exploration towards a novel over a familiar object [39]. Following 18 days of pravastatin treatment, rats were significantly impaired in this task. The pravastatin treated animals spent relatively less time exploring the novel object when compared with the control treated group suggesting they were less able to discriminate novelty following a 4-hour delay period. These deficits were fully reversed when animals were re-tested 10 days after the end of treatment, confirming that deficits were temporary. Similar to the data from the SDRL task, atorvastatin-treated animals were not significantly different from control animals. Impaired recognition memory in rodents is used as a model for human cognitive dysfunction and has been demonstrated using manipulations which inhibit short and long term memory formation. A number of studies have shown that object recognition memory is significantly disrupted by lesions in the rat perirhinal cortex [40–42],, as well as by local inhibition of cholinergic neurotransmission [43], and cell signalling pathways involved in synaptic plasticity, such as CAMKK [44].

In this study we observed no effects of atorvastatin treatment on memory function. Only a single dose of each drug was tested and this may account for the lack of effect with atorvastatin, however these data do suggest that the effects are not dependent on the lipophilicity of the drug. Atorvastatin is a relatively lipophilic compound whilst pravastatin is more hydrophilic due to the presence of a polar hydroxyl group [45], and some studies suggest little, if any, pravastatin crosses the blood–brain barrier [46]. However, pravastatin has been detected within the cerebral cortex of chronically treated mice at levels above the IC_50_ of HMG-CoA reductase activity [47]. It is therefore possible that despite its hydrophilicity, pravastatin may still be able to pass into the central nervous system. Organic anion transporters (OATS) expressed in the brain have been shown to transport pravastatin [48,49], and it has been found that chronic pravastatin treatment in mice increases cortical gene expression of the monocarboxylic acid transporter (*MCT2*), which transports statin acids [47,50]. Although atorvastatin is more potent in its hypocholesterolaemic effects relative to pravastatin [51], a recent clinical study demonstrated that intake of statins, independent of their lipophilicity, led to significantly lower levels of lathosterol (an indicator of cholesterol synthesis) in the cerebrospinal fluid [22]. Therefore it may be that the difference in effects of the statins on cognitive function observed in this study is independent of their lipophilicity.

Research into the effects of chronic statin treatment on cognitive function in animals has yielded conflicting results. Atorvastatin was found to improve consolidation of spatial memory in mice, an effect dependent on nitric oxide signalling pathways [28]. Similarly, chronic treatment with simvastatin over 25 days improved the performance of normal rats in an object-in-place recognition memory task [29]. However, clinical trials have raised the possibility that prolonged statin use may not be beneficial in terms of improving cognitive function in humans, with the large-scale Prospective Study of Pravastatin in the Elderly at Risk (PROSPER) trial showing no effect of pravastatin on cognitive function [52]. Furthermore, a recent patient survey-based analysis of statin-associated adverse drug reactions has reported drug-induced cognitive problems that resolve after treatment discontinuation [15], supporting the findings of the present study that chronic statin treatment can have adverse cognitive side effects.

The mechanism by which statins might worsen cognitive functions is unknown, but one of the prevailing hypotheses focuses on the role of cholesterol in the brain. Since cholesterol plays a fundamental role in the myelination of neurons, it has been proposed that excessive inhibition of cholesterol synthesis could lead to adverse cognitive effects [5,6,22]. It is thought that brain cholesterol is mainly formed by *in situ* synthesis and the blood–brain barrier effectively separates this and other compounds from the periphery [25]. Indeed, data from animal studies suggest that the brain has a remarkable capacity to maintain cholesterol homeostasis in spite of marked changes in systemic cholesterol levels [25,26]. However, there is some evidence that statin treatment inhibits local synthesis of cholesterol in the CNS. Lütjohann et al. (2004) [25] observed a small but significant decrease in de novo cholesterol synthesis in the brains of guinea pigs after 3 weeks of treatment with pravastatin. Similarly, Vecka et al. (2004) [24] reported that pravastatin lowers brain cholesterol following chronic dosing in rats. Given the important role for brain lipids in normal brain function, reduced local synthesis of cholesterol may represent a mechanism through which the cognitive effects of pravastatin treatment may be mediated.

In addition to their established role in the lowering of circulating chloresterol, statins have been shown to influence a number of cellular functions [53]. Statin-induced changes in cellular activity have mostly been related to disruption of Ras-mediated signalling [54], which plays an important role in synaptic events leading to memory consolidation [55]. Statins also target the Rab-dependent pathway of receptor endocytosis, which functions as a key regulator of intracellular trafficking [56]. Other research groups have attributed the cellular functions of statins to alterations in the cholesterol-rich membrane microdomains known as ‘lipid rafts’, and lowering cholesterol levels in vitro has been shown to disrupt the stability of these rafts [57]. The resulting disruption in cell signalling may contribute to cognitive impairment associated with statin treatment [58].

In order to further investigate possible reasons for the differences in effects seen with pravastatin versus atorvastatin in our experiment, we compared their chemical, pharmacokinetic and pharmacodynamic properties [58]. Pravastatin is a fungal-derived HMG-CoA reductase inhibitor whilst atorvastatin is fully synthetic, and structural differences between the two compounds are known to influence their pharmacokinetic profile [58]. Atorvastatin has a much longer half-life in man than pravastatin and is subject to CYP450 metabolism, whilst pravastatin is excreted largely unchanged [58,59]. Atorvastatin is highly plasma protein bound and has an active metabolite whilst pravastatin is the least protein bound of any statin (~50%) and has a much shorter half life, although both drugs are proposed to have similar bioavailability [58,59]. The drugs also have fairly similar efficacies in terms of activity at the enzyme with IC_50_ values in primary rat hepatocytes 1.16nM for atorvastatin and 6.93nM for pravastatin [60]. Where a major difference exists is the plasma levels of the two drugs. For the majority of statins, including atorvastatin, efficient first pass uptake into the liver occurs and has been seen as a benefit to the effects of statins given that the liver is the major target organ [58]. This pharmacokinetic property also limits the amount of drug in the general circulation. Because of its low first pass metabolism and estimated lower plasma protein binding, pravastatin is found in much higher levels in plasma and is therefore much more widely distributed than the other statin drugs [59]. Although the hydrophillic nature of the drug is thought to limit its effects on other organs, this much wider distribution is a clear difference between the two statins tested in this study and may lead to a greater ability to affect the CNS. Pertinent to our observation that lipophilicity was not related to the CNS effects observed here, is a study into the effects of different statins in Alzheimer’s disease which also found a lack of correlation with lipophilicity [61].

In conclusion, we have demonstrated that chronic treatment with pravastatin impairs recognition memory in normal rats. We also found impairments in working memory towards the end of treatment although this effect was less clear and was limited by the duration of treatment used in this study. Both effects observed were fully reversed following cessation of treatment. Overall, these findings support clinical observations that statins have adverse cognitive effects in certain patients undergoing long-term treatment. This study provides some of the first data obtained from animal studies suggesting statin treatment can impair cognitive function in a reversible manner in normal animals. The deficits observed with pravastatin were present in both models suggesting impaired working memory and/or attention, impaired cognitive flexibility and impaired recognition memory. Although this study was limited to a single dose of each statin and only two statins were tested, the reversibility of the deficits observed support a drug-specific mechanism. It is clear that further studies are needed to elucidate the mechanisms involved in the effects observed including looking at a wider dose range and additional types of statin treatment. It is also necessary to now extend this work to look at the relationship between cognitive effects and direct measurements of plasma, brain and liver cholesterol and how this relates to drug distribution and CNS side effects. All statins have been reported to potentially affect cognition and the whole class carry an FDA warning. Our data suggest that more detailed, pre-clinical studies to investigate different types of statins and cognitive function, including a wider range of doses of the drugs tested here, would be useful in determining the mechanisms involved and relative risks associated with different statin treatments.

## References

[1] Hsu I, Spinler SA, Johnson NE (1995) Comparative evaluation of the safety and efficacy of HMG-CoA reductase inhibitor monotherapy in the treatment of primary hypercholesterolemia. Ann Pharmacother 29: 743-759. PubMed: 8520093. 8520093 10.1177/106002809502907-818

[2] Davidson MH (2001) Safety profiles for the HMG-CoA reductase inhibitors: treatment and trust. Drugs 61: 197-206. doi:10.2165/00003495-200161020-00005. PubMed: 11270938. 11270938 10.2165/00003495-200161020-00005

[3] Dales M (2000) Statination. Intern Med NEWS Vol. 1: 55–56.

[4] Dietschy JM, Turley SD (2001) Cholesterol metabolism in the brain. Curr Opin Lipidol 12: 105-112. doi:10.1097/00041433-200104000-00003. PubMed: 11264981. 11264981 10.1097/00041433-200104000-00003

[5] Pfrieger FW (2003) Role of cholesterol in synapse formation and function. Biochim Biophys Acta 1610: 271-280. doi:10.1016/S0005-2736(03)00024-5. PubMed: 12648780. 12648780 10.1016/s0005-2736(03)00024-5

[6] Pfrieger FW, Ungerer N (2011) Cholesterol metabolism in neurons and astrocytes. Prog Lipid Res 50: 357-371. doi:10.1016/j.plipres.2011.06.002. PubMed: 21741992. 21741992 10.1016/j.plipres.2011.06.002

[7] FDA (2012) Drug Safety Communication: Important safety label changes to cholesterol-lowering statin drugs. http://www.fda.gov/Drugs/DrugSafety/ucm293101.htm.

[8] Etminan M, Gill S, Samii A (2003) The role of lipid-lowering drugs in cognitive function: a meta-analysis of observational studies. Pharmacotherapy 23: 726-730. doi:10.1592/phco.23.6.726.32184. PubMed: 12820814. 12820814 10.1592/phco.23.6.726.32184

[9] Jick H, Zornberg GL, Jick SS, Seshadri S, Drachman DA (2000) Statins and the risk of dementia. Lancet 356: 1627-1631. doi:10.1016/S0140-6736(00)03155-X. PubMed: 11089820. 11089820 10.1016/s0140-6736(00)03155-x

[10] Wolozin B, Kellman W, Ruosseau P, Celesia GG, Siegel G (2000) Decreased prevalence of Alzheimer disease associated with 3-hydroxy-3-methyglutaryl coenzyme A reductase inhibitors. Arch Neurol 57: 1439-1443. doi:10.1001/archneur.57.10.1439. PubMed: 11030795. 11030795 10.1001/archneur.57.10.1439

[11] Feldman HH, Doody RS, Kivipelto M, Sparks DL, Waters DD et al. (2010) Randomized controlled trial of atorvastatin in mild to moderate Alzheimer disease: LEADe. Neurology 74: 956-964. doi:10.1212/WNL.0b013e3181d6476a. PubMed: 20200346. 20200346 10.1212/WNL.0b013e3181d6476a

[12] King DS, Wilburn AJ, Wofford MR, Harrell TK, Lindley BJ et al. (2003) Cognitive impairment associated with atorvastatin and simvastatin. Pharmacotherapy 23: 1663-1667. doi:10.1592/phco.23.15.1663.31953. PubMed: 14695047. 14695047 10.1592/phco.23.15.1663.31953

[13] Padala KP, Padala PR, Potter JF (2006) Simvastatin-induced decline in cognition. Ann Pharmacother 40: 1880-1883. doi:10.1345/aph.1H014. PubMed: 16940411. 16940411 10.1345/aph.1H014

[14] Muldoon MF, Ryan CM, Sereika SM, Flory JD, Manuck SB (2004) Randomized trial of the effects of simvastatin on cognitive functioning in hypercholesterolemic adults. Am J Med 117: 823-829. doi:10.1016/j.amjmed.2004.07.041. PubMed: 15589485. 15589485 10.1016/j.amjmed.2004.07.041

[15] Evans MA, Golomb BA (2009) Statin-associated adverse cognitive effects: survey results from 171 patients. Pharmacotherapy 29: 800-811. doi:10.1592/phco.29.7.800. PubMed: 19558254. 19558254 10.1592/phco.29.7.800

[16] Abrahamson EE, Ikonomovic MD, Dixon CE, DeKosky ST (2009) Simvastatin therapy prevents brain trauma-induced increases in beta-amyloid peptide levels. Ann Neurol 66: 407-414. doi:10.1002/ana.21731. PubMed: 19798641. 19798641 10.1002/ana.21731

[17] Wang H, Lynch JR, Song P, Yang HJ, Yates RB et al. (2007) Simvastatin and atorvastatin improve behavioral outcome, reduce hippocampal degeneration, and improve cerebral blood flow after experimental traumatic brain injury. Exp Neurol 206: 59-69. doi:10.1016/j.expneurol.2007.03.031. PubMed: 17521631. 17521631 10.1016/j.expneurol.2007.03.031

[18] Lu D, Qu C, Goussev A, Jiang H, Lu C et al. (2007) Statins increase neurogenesis in the dentate gyrus, reduce delayed neuronal death in the hippocampal CA3 region, and improve spatial learning in rat after traumatic brain injury. J Neurotrauma 24: 1132-1146. doi:10.1089/neu.2007.0288. PubMed: 17610353. 17610353 10.1089/neu.2007.0288 PMC1971229

[19] Mans RA, Chowdhury N, Cao D, McMahon LL, Li L (2010) Simvastatin enhances hippocampal long-term potentiation in C57BL/6 mice. Neuroscience 166: 435-444. doi:10.1016/j.neuroscience.2009.12.062. PubMed: 20040368. 20040368 10.1016/j.neuroscience.2009.12.062 PMC2824052

[20] Wu H, Lu D, Jiang H, Xiong Y, Qu C et al. (2008) Simvastatin-mediated upregulation of VEGF and BDNF, activation of the PI3K/Akt pathway, and increase of neurogenesis are associated with therapeutic improvement after traumatic brain injury. J Neurotrauma 25: 130-139. doi:10.1089/neu.2007.0369. PubMed: 18260796. 18260796 10.1089/neu.2007.0369

[21] Baytan SH, Alkanat M, Okuyan M, Ekinci M, Gedikli E et al. (2008) Simvastatin impairs spatial memory in rats at a specific dose level. Tohoku J Exp Med 214: 341-349. doi:10.1620/tjem.214.341. PubMed: 18441510. 18441510 10.1620/tjem.214.341

[22] Fassbender K, Stroick M, Bertsch T, Ragoschke A, Kuehl S et al. (2002) Effects of statins on human cerebral cholesterol metabolism and secretion of Alzheimer amyloid peptide. Neurology 59: 1257-1258. doi:10.1212/WNL.59.8.1257. PubMed: 12391360. 12391360 10.1212/wnl.59.8.1257

[23] Oldfield S, Hancock J, Mason A, Hobson SA, Wynick D et al. (2009) Receptor-mediated suppression of potassium currents requires colocalization within lipid rafts. Mol Pharmacol 76: 1279-1289. doi:10.1124/mol.109.058008. PubMed: 19726551. 19726551 10.1124/mol.109.058008

[24] Vecka M, Tvrzická E, Stanková B, Novák F, Nováková O et al. (2004) Hypolipidemic drugs can change the composition of rat brain lipids. Tohoku J Exp Med 204: 209-308. doi:10.1620/tjem.204.209. PubMed: 15502420. 15572855 10.1620/tjem.204.299

[25] Lütjohann D, Stroick M, Bertsch T, Kühl S, Lindenthal B et al. (2004) High doses of simvastatin, pravastatin, and cholesterol reduce brain cholesterol synthesis in guinea pigs. Steroids 69: 431-438. doi:10.1016/j.steroids.2004.03.012. PubMed: 15219793. 15219793 10.1016/j.steroids.2004.03.012

[26] Thelen KM, Rentsch KM, Gutteck U, Heverin M, Olin M et al. (2006) Brain cholesterol synthesis in mice is affected by high dose of simvastatin but not of pravastatin. J Pharmacol Exp Ther 316: 1146-1152. PubMed: 16282522. 16282522 10.1124/jpet.105.094136

[27] Kilkenny C, Browne W, Cuthill IC, Emerson M, Altman DG (2010) Animal research: Reporting in vivo experiments: The ARRIVE guidelines. Br J Pharmacol 160: 1577-1579. doi:10.1111/j.1476-5381.2010.00872.x. PubMed: 20649561. 20649561 10.1111/j.1476-5381.2010.00872.x PMC2936830

[28] Javadi-Paydar M, Rayatnia F, Fakhraei N, Zakeri M, Mirazi NA et al. (2011) Atorvastatin improved scopolamine-induced impairment in memory acquisition in mice: involvement of nitric oxide. Brain Res 1386: 89-99. doi:10.1016/j.brainres.2011.02.057. PubMed: 21354117. 21354117 10.1016/j.brainres.2011.02.057

[29] Douma TN, Borre Y, Hendriksen H, Olivier B, Oosting RS (2011) Simvastatin improves learning and memory in control but not in olfactory bulbectomized rats. Psychopharmacology (Berl) 216: 537-544. doi:10.1007/s00213-011-2245-0. 21384104 10.1007/s00213-011-2245-0 PMC3140942

[30] Shepherd SJ, Hunninghake DB, Barter P, McKenney JM, Hutchinson HG (2003) Guidelines for lowering lipids to reduce coronary artery disease risk: a comparison of rosuvastatin with atorvastatin, pravastatin, and simvastatin for achieving lipid-lowering goals. Am J Cardiol 91(5A): 11C–17C. PubMed: 12646338. 10.1016/s0002-9149(03)00004-3 12646338

[31] Zhou Z, Rahme E, Pilote L (2006) Are statins created equal? Evidence from randomized trials of pravastatin, simvastatin, and atorvastatin for cardiovascular disease prevention. Am Heart J, 151: 273–281. doi:10.1016/j.ahj.2005.04.003. PubMed: 16442888. 16442888 10.1016/j.ahj.2005.04.003

[32] Birrell JM, Brown VJ (2000) Medial frontal cortex mediates perceptual attentional set shifting in the rat. J Neurosci 20: 4320-4324. PubMed: 10818167. 10818167 10.1523/JNEUROSCI.20-11-04320.2000 PMC6772641

[33] Idris NF, Repeto P, Neill JC, Large CH (2005) Investigation of the effects of lamotrigine and clozapine in improving reversal-learning impairments induced by acute phencyclidine and D-amphetamine in the rat. Psychopharmacology (Berl) 179: 336-348. doi:10.1007/s00213-004-2058-5. PubMed: 15645224. 15645224 10.1007/s00213-004-2058-5

[34] Jentsch JD, Taylor JR (2001) Impaired inhibition of conditioned responses produced by subchronic administration of phencyclidine to rats. Neuropsychopharmacology 24: 66-74. doi:10.1016/S0893-133X(00)00174-3. PubMed: 11106877. 11106877 10.1016/S0893-133X(00)00174-3

[35] Jones GH, Marsden CA, Robbins TW (1991) Behavioural rigidity and rule-learning deficits following isolation-rearing in the rat: neurochemical correlates. Behav Brain Res 43: 35-50. doi:10.1016/S0166-4328(05)80050-6. PubMed: 1677579. 1677579 10.1016/s0166-4328(05)80050-6

[36] Robbins TW (2002) The 5-choice serial reaction time task: behavioural pharmacology and functional neurochemistry. Psychopharmacology (Berl) 163: 362-380. doi:10.1007/s00213-002-1154-7. PubMed: 12373437. 12373437 10.1007/s00213-002-1154-7

[37] Mackintosh N, Little L (1969) Selective attention and response strategies as factors in serial reversal learning. Can J Psychol 23: 335–346. doi:10.1037/h0082821.

[38] Aggleton JP, Hunt PR, Rawlins JN (1986) The effects of hippocampal lesions upon spatial and non-spatial tests of working memory. Behav Brain Res 19: 133-146. doi:10.1016/0166-4328(86)90011-2. PubMed: 3964405. 3964405 10.1016/0166-4328(86)90011-2

[39] Ennaceur A, Delacour J (1988) A new one-trial test for neurobiological studies of memory in rats. 1: Behavioral data. Behav Brain Res 31: 47-59. doi:10.1016/0166-4328(88)90157-X. PubMed: 3228475. 3228475 10.1016/0166-4328(88)90157-x

[40] Barker GR, Bird F, Alexander V, Warburton EC (2007) Recognition memory for objects, place, and temporal order: a disconnection analysis of the role of the medial prefrontal cortex and perirhinal cortex. J Neurosci 27: 2948-2957. doi:10.1523/JNEUROSCI.5289-06.2007. PubMed: 17360918. 17360918 10.1523/JNEUROSCI.5289-06.2007 PMC6672574

[41] Mumby DG, Piterkin P, Lecluse V, Lehmann H (2007) Perirhinal cortex damage and anterograde object-recognition in rats after long retention intervals. Behav Brain Res 185: 82-87. doi:10.1016/j.bbr.2007.07.026. PubMed: 17804090. 17804090 10.1016/j.bbr.2007.07.026

[42] Winters BD, Forwood SE, Cowell RA, Saksida LM, Bussey TJ (2004) Double dissociation between the effects of peri-postrhinal cortex and hippocampal lesions on tests of object recognition and spatial memory: heterogeneity of function within the temporal lobe. J Neurosci 24: 5901-5908. doi:10.1523/JNEUROSCI.1346-04.2004. PubMed: 15229237. 15229237 10.1523/JNEUROSCI.1346-04.2004 PMC6729235

[43] Warburton EC, Koder T, Cho K, Massey PV, Duguid G et al. (2003) Cholinergic neurotransmission is essential for perirhinal cortical plasticity and recognition memory. Neuron 38: 987-996. doi:10.1016/S0896-6273(03)00358-1. PubMed: 12818183. 12818183 10.1016/s0896-6273(03)00358-1

[44] Tinsley CJ, Narduzzo KE, Brown MW, Warburton EC (2012) A role for the CAMKK pathway in visual object recognition memory. Hippocampus 22: 466-476. doi:10.1002/hipo.20913. PubMed: 21298728. 21298728 10.1002/hipo.20913

[45] McTavish D, Sorkin EM (1991) Pravastatin. A review of its pharmacological properties and therapeutic potential in hypercholesterolaemia. Drugs 42: 65-89. doi:10.2165/00003495-199142010-00005. PubMed: 1718686. 1718686 10.2165/00003495-199142010-00005

[46] Botti RE, Triscari J, Pan HY, Zayat J (1991) Concentrations of pravastatin and lovastatin in cerebrospinal fluid in healthy subjects. Clin Neuropharmacol 14: 256-261. doi:10.1097/00002826-199106000-00010. PubMed: 1906375. 1906375 10.1097/00002826-199106000-00010

[47] Johnson-Anuna LN, Eckert GP, Keller JH, Igbavboa U, Franke C et al. (2005) Chronic administration of statins alters multiple gene expression patterns in mouse cerebral cortex. J Pharmacol Exp Ther 312: 786-793. PubMed: 15358814. 15358814 10.1124/jpet.104.075028

[48] Kusuhara H, Sekine T, Utsunomiya-Tate N, Tsuda M, Kojima R et al. (1999) Molecular cloning and characterization of a new multispecific organic anion transporter from rat brain. J Biol Chem 274: 13675-13680. doi:10.1074/jbc.274.19.13675. PubMed: 10224140. 10224140 10.1074/jbc.274.19.13675

[49] Takeda M, Noshiro R, Onozato ML, Tojo A, Hasannejad H et al. (2004) Evidence for a role of human organic anion transporters in the muscular side effects of HMG-CoA reductase inhibitors. Eur J Pharmacol 483: 133-138. doi:10.1016/j.ejphar.2003.10.017. PubMed: 14729100. 14729100 10.1016/j.ejphar.2003.10.017

[50] Tsuji A, Saheki A, Tamai I, Terasaki T (1993) Transport mechanism of 3-hydroxy-3-methylglutaryl coenzyme A reductase inhibitors at the blood-brain barrier. J Pharmacol Exp Ther 267: 1085-1090. PubMed: 8263769. 8263769

[51] Jones P, Kafonek S, Laurora I, Hunninghake D (1998) Comparative dose efficacy study of atorvastatin versus simvastatin, pravastatin, lovastatin, and fluvastatin in patients with hypercholesterolemia (the CURVES study). Am J Cardiol 81: 582-587. doi:10.1016/S0002-9149(97)00965-X. PubMed: 9514454. 9514454 10.1016/s0002-9149(97)00965-x

[52] Shepherd J, Blauw GJ, Murphy MB, Bollen EL, Buckley BM et al. (2002) Pravastatin in elderly individuals at risk of vascular disease (PROSPER): a randomised controlled trial. Lancet 360: 1623-1630. doi:10.1016/S0140-6736(02)11600-X. PubMed: 12457784. 12457784 10.1016/s0140-6736(02)11600-x

[53] Takemoto M, Liao JK (2001) Pleiotropic effects of 3-hydroxy-3-methylglutaryl coenzyme a reductase inhibitors. Arterioscler Thromb Vasc Biol 21: 1712-1719. doi:10.1161/hq1101.098486. PubMed: 11701455. 11701455 10.1161/hq1101.098486

[54] Van Aelst L, D’Souza-Schorey C (1997) Rho GTPases and signaling networks. Genes Dev 11: 2295-2322. doi:10.1101/gad.11.18.2295. PubMed: 9308960. 9308960 10.1101/gad.11.18.2295

[55] Brambilla R, Gnesutta N, Minichiello L, White G, Roylance AJ et al. (1997) A role for the Ras signalling pathway in synaptic transmission and long-term memory. Nature 390: 281-286. doi:10.1038/36849. PubMed: 9384379. 9384379 10.1038/36849

[56] Ghittoni R, Napolitani G, Benati D, Ulivieri C, Patrussi L et al. (2006) Simvastatin inhibits the MHC class II pathway of antigen presentation by impairing Ras superfamily GTPases. Eur J Immunol 36: 2885-2893. doi:10.1002/eji.200636567. PubMed: 17048274. 17048274 10.1002/eji.200636567

[57] Hering H, Lin CC, Sheng M (2003) Lipid rafts in the maintenance of synapses, dendritic spines, and surface AMPA receptor stability. J Neurosci 23: 3262-3271. PubMed: 12716933. 12716933 10.1523/JNEUROSCI.23-08-03262.2003 PMC6742299

[58] Schachter M (2004) Chemical, pharmacokinetic and pharmacodynamic properties of statins: an update. Fundam Clin Pharmacol 19: 117-125. PubMed: 15660968. 10.1111/j.1472-8206.2004.00299.x 15660968

[59] Rosenson RS (2003) Rosuvastatin: a new inhibitor of HMG-CoA reductase for the treatment of dyslipidemia. Expert Rev Cardiovasc Ther 1: 495-505. doi:10.1586/14779072.1.4.495. PubMed: 15030249. 15030249 10.1586/14779072.1.4.495

[60] McTaggart F, Buckett L, Davidson R (2001) Preclinical and clinical pharmacology of rosuvastatin, a new 3-hydroxyl-3methylglutaryl coenzyme A reductase inhibitor. Am J Cardiol 87: 28-32. doi:10.1016/S0002-9149(01)01719-2. PubMed: 11137829. 11256847 10.1016/s0002-9149(01)01454-0

[61] Haag MDM, Hofman A, Koudstaal PJ, Stricker BHC, Breteler MMB (2009) Statins are associated with a reduced risk of Alzheimer disease regardless of lipophilicity. The Rotterdam Study J. Neurol Neurosurg Psychiatry 80: 13-17. doi:10.1136/jnnp.2008.150433. 10.1136/jnnp.2008.150433 18931004

